# The Neuronal Cotransmission: Mechanistic Insights From the Autonomic Nervous System

**DOI:** 10.7759/cureus.35124

**Published:** 2023-02-17

**Authors:** Hussain Aldera, Omar A AlQahtani, Mushabab A AlQahtani, Saad M Al Nasher, Muhannad Q Alqirnas

**Affiliations:** 1 Neurophysiology, Department of Basic Medical Sciences, College of Medicine, King Saud bin Abdulaziz University for Health Sciences, Riyadh, SAU; 2 College of Medicine, King Khalid University, Riyadh, SAU; 3 College of Medicine, King Khalid University, Abha, SAU; 4 Medicine, College of Medicine, King Saud Bin Abdulaziz University for Health Sciences, Riyadh, SAU

**Keywords:** cotransmitter hypothesis, cotransmission synergy, autonomic nervous system, neuronal cotransmission, cotransmission

## Abstract

It is now scientifically* *accepted that neurons have the ability to release multiple transmitter substances simultaneously, yet, cotransmission's functionality is still limited to the scientific community. Acetylcholine is released by the noradrenergic neurons, and then the acetylcholine works prejunctionally in the promotion of the noradrenaline release. This hypothesis significantly challenged the previous idea of autonomic transmission as being a simple process that had a single transmitter. Norepinephrine was thought to be the single transmitter at the sympathetic neurovascular junction according to “Dale’s principle". However, more evidence of the involvement of other neurotransmitters has been shown by many researchers in conjunction with Dale's principle and established terms such as adrenergic, purinergic, and peptidergic nerves. With the discovery of cotransmission, we now understand the existence of more than one neurotransmitter at a sympathetic neurovascular junction.

## Introduction and background

Transmission at the sympathetic neuroeffector junction

Neurovascular transmission refers to the communication between the nerves which supply the blood vessels and the blood vessel itself. It is where the blood vessels can receive signals from the nerves, which innervate them to produce a change in the tone of the blood vessel [1]. Norepinephrine was thought to be the single transmitter at the sympathetic neurovascular junction according to “Dale’s principle". It stated that a certain neuron would have the same effect on all the connections it has with any other form of a cell that the nerve is innervating regardless of the type of tissue of that cell [2,3]. However, more evidence to the involvement of other neurotransmitters has been shown by many researchers in conjunction with Dale's principle and established terms such as adrenergic, purinergic, and peptidergic nerves [4-6]. These separate nerves described the neuroanatomy of the vasculature, so they became accepted. However, with the discovery of cotransmission, we now understand the existence of more than one neurotransmitter at a sympathetic neurovascular junction.

## Review

Concept of cotransmission

A cotransmitter is a substance that transmits messages to the cells that surround it or to the same nerve when the nerve releases it, hence modulating their roles. Cotransmission therefore can be defined as the releasing of a substance (cotransmitter) together with primary neurotransmitter from the nerve endings to modify the cotransmitter. For instance, the vasoactive intestinal peptide works like a cotransmitter with the acetylcholine in the cholinergic synapses [7]. Researchers, even as early as the 1970s, have always considered cotransmission as an extreme curiosity and which plays a given role in the unusual nerve stimulation responses visible in some smooth muscles which are innervated autonomically. Various researchers have agreed with cotransmission as a rule and not the neurotransmission exception [8].

Early evidence

Between 1950 and 1960, work was done on cotransmission in the autonomic nerves. Rand and Burn did this stimulating work, and it provided a false start to the cotransmission concept. According to them, acetylcholine was released by the noradrenergic neurons, and then the acetylcholine worked prejunctionally in the promotion of the noradrenaline release [9]. Even though it is no longer viewed as reasonable, this hypothesis significantly challenged the previous idea of autonomic transmission as being a simple process that had a single transmitter.

Around the 1960s, there was the availability of selective as well as new drugs with the autonomic neurotransmission pharmacology undergoing deep investigation. Burnstock and his Australian team [10] realized that responses of several preparations of the smooth muscles that were isolated could not have a clear explanation on the basis of classical noradrenergic as well as cholinergic mechanisms thereby using non-adrenergic, non-cholinergic (NANC) nerves in indication of the current autonomic nerve class. They then worked on identifying the neurotransmitter of NANC, which they identified as the purine adenosine 5-triphosphate (ATP) around the 1970s. The review of the topic, which contained evidence for the support of purinergic nerves, was later presented in 1972. Therefore the hypothesis of the purinergic nerves played a very significant role in reinforcing investigations on the idea of autonomic nerve cotransmission [10].

Cotransmitter hypothesis

The early cotransmission proof happened during the time when different studies doing the investigation of the isolated smooth muscles' mechanical responses to the NANC nerve stimulation started being reassessed based on the cotransmission possibility. Isolated vas deferens from rodents were among the most important preparations which unveiled more details on cotransmission.

The result from various vas deferens studies with details on the mechanical responses made the evidence even stronger, strengthening the idea that the ATP and noradrenaline function as cotransmitters in mediating the muscle’s complicated mechanical responses to its sympathetic innervation stimulation. In the sympathetic nerves, ATP has also been proven to be a cotransmitter with noradrenalin while acting the same role on the parasympathetic nerves using acetylcholine, and this happens in several tissues that are autonomically innervated, for instance, the urinary bladder's smooth muscle and also in the smooth muscle of the vascular. Currently, there is substantial evidence of the putative cotransmitter colocalization in the central nervous system, nerves of the somatic motor, peripheral autonomic ganglia, and the sensory neurons, both in mammals as well as invertebrates, amphibians, crustaceans, birds, and insects [11].

Synthesis, storage, and release

Blaschko and his team [12] did some work in the early 1950s which revealed noradrenaline's synthetic pathway and the high-concentration existence of ATP, which is also stored with the noradrenaline in the adrenal medulla's chromaffin granules. It was also indicated that the vesicles of the noradrenergic nerves were not only able to store but also release ATP, noradrenaline included [13]. This was not considered evidence until later on. Many researchers have always regarded the ATP presence as the substance for the packaging and also storage of noradrenaline within the sympathetic vesicle. There exist various biochemical estimations of the quantity of noradrenaline to the ATP, which is stored in the sympathetic vesicles, with ranges from 4:1 to the most current and reliable estimation of 20:1 up to 50:1. After the discovery of the ATP as the noradrenergic nerve terminal constituent, it became clear that its storage was also together with the acetylcholine in the terminals of the cholinergic nerves [14].

During the 1970s, due to considerable impetus on cotransmission, there were significant advances since the immunochemical methods were applied in the localization of the number of putative peptide neurotransmitters that were continuously increasing. Currently, multiple researches in the literature document many examples of peptide colocalization in all neurons [15]. However, a consistent pattern giving more insight into the importance of functions on the various peptide coexistences has been found.

The co-storage of a single or several peptides together with the non-peptide neurotransmitters seem like a vital combination. Neuropeptide Y (NPY) is a better example of this, considering that it is among the neuropeptides which are abundant and found within the central nervous system as well as the peripheral neurons, where it exists with noradrenaline in combination [16]. However, there is the coexistence of noradrenaline and ATP (non-peptide neurotransmitters) in the vas deferens of guinea pig [17,18]. The neocortical neurons also have vasoactive intestinal peptides and acetylcholine in the central nervous system. There is some functional significance in the idea that while the synthesis of purine ATP, acetylcholine, and noradrenalin happens locally in the nerve terminals, the production of the neuropeptide always gets restricted to ribosomes in the body of the nerve cells. The peptide then gets transported in the vesicles from cell soma to nerve terminals through axonal transport, which is a slow process. This signifies that there is some little amount of peptide which could be readily released, while the stores will take a considerably longer time to replace after nerve activity burst. There is an indication by biochemical estimations that the concentration of the peptides that are stored in nerves are many orders of the magnitude, which is lower than classical cotransmitter concentration. The peptides, therefore, generally seem to have a high receptor affinity, functioning at a very low concentration compared to the conventional cotransmitters, mainly becoming active in the nanomolar range, compared with micromolar concentrations, which are associated with the non-peptide transmitters [19].

Vas deferens of rodents as a differentiating model for the transmitter postjunctional actions

A significant characteristic relevant to the vas deferens functioning is the consideration that the cells of the smooth muscles in various tissue regions tend to own some noradrenaline and purine ATP sensitivities. They have also been recent indications that vas deferens segments extracted from a rat's, guinea pig’s, or rabbit's prostatic vas deferens region are almost 10 times as sensitive compared to the epididymal segments to the exogenous purine ATP, with the epididymal region’s segments ten times more sensitive in comparison to the prostatic segments to the application of the agonists of the exogenous adrenoceptor like the noradrenalin [9,17].

The first evidence for the concept of transmission involving ATP was in early 1971 in California through a study by Su and colleagues on guinea pig. In this study, Su and colleagues have used the isotopes technique to examine a hypothesis that ATP is the transmitter substance released by non-adrenergic inhibitory nerves in the gut, by measuring the uptake of tritium-labelled nucleosides and nucleotides, their conversion and storage in the tissue, and their release during enteric nerve stimulation in the taenia coli of the guinea pig. Taenia coli were selected by the authors because the radioactivity released during inhibitory responses to both adrenergic and non-adrenergic nerves could be monitored and comparable. They came up in uptake study analysis that concentration of tritiated inosine and adenine (the metabolites of ATP) with a much smaller degree. Furthermore, this ATP in the release analysis part of the experiment was released upon activation of both adrenergic and non-adrenergic inhibitory nerves. This was the first evidence for the concept cotransmission principle that might exist [20].

An application of ATP caused a "twitch"-like contraction with very short latency (within one second) of the guinea pig seminal vesicle. However, the amplitude of contraction made by ATP was less than that of the nerve-mediated contraction. This was a significant observation by Nakanishi and Takeda [21], who had conducted a study that concluded that ATP has a considerable role in addition to norepinephrine (NE) in the excitatory transmission between the hypogastric nerve terminal and seminal vesicle in the guinea pig.

It is worth saying that ATP is released from adrenergic nerves simultaneously with norepinephrine. This was not just a hypothesis anymore because it was proven indirectly by Su and others [19] when he suggested that ATP might act as inhibitory modulators in relationship with norepinephrine (adrenergic nerve endings). This encouraged Westfall and his colleagues [22] to conduct a study to examine the post-junctional effects and neural release on purine compounds in the guinea pig vas deferens. The smooth muscle of the guinea pig vas deferens in vitro was shown to contract upon the addition of ATP. Administering the tissues with α-adrenoceptor blocking agent failed to change the dose-response relationship for ATP. Since ATP is considered a potent contractile mediator [19] and is stored in the complex adrenergic vesicle [23], Westfall and his colleagues [22] then experimented using a group of vas deferens of guinea pigs which were set up in isolated organ baths and applied resting tension to them. ATP, adenosine, and adenosine-5-diphosphate (ADP) were tested to check in vitro their ability to contract the vas deferens. Isometric contractions of the smooth muscle were recorded by polygraph. Dose-response curves were attained by increasing in concentration of the agonist. Then, the tissues were inserted into a pair of electrodes to facilitate either transmural electrical or drug-induced stimulation. Tetrodotoxin (TTX) as a drug was used in order to abolish the release of 3H noradrenaline. They concluded that the response of the smooth muscles vas deferens to ATP consisted of a rapid and phasic contraction. However, this contraction induced by ATP was not reliant on endogenous noradrenaline as a result of the elimination of the action of norepinephrine by reserpine. Also, they showed that nerve stimulation made the TTX more sensitive to the release of tritium in the tissues of 3H-adenosine even without contraction. Previous studies stated that in vas deferens, about 90-98% of the total tissue radioactivity were 3H-nucleotides. Westfall and his colleagues strongly suggested that ATP is released with noradrenaline as a cotransmitter, not a modulator.

In the vas deferens of rats, smooth muscle's biphasic contraction is formed by a single sympathetic nerve stimulus. The purine ATP mediates the first peak, while noradrenaline mediates the second [24,25]. Pharmacological investigations using selective antagonists have presented thorough arguments supporting this interpretation [26]. Agents inhibiting the purine ATP actions on the P2 purinoceptors, like suramin, selectively reduce the initial phase responses in guinea pigs vas deferens [27] and rat tail arteries [28]. α-adrenoceptor antagonist selectively reduces the second phase [29]. The reason for the resistant action of this first phase contractile response in vas deferens to prazosin or any α-adrenoceptor antagonists encouraged many scientists to think that there might be another transmitter caused this contraction noradrenaline for example, McGrath and O'Brien [30] suggested that there might be another neurotransmitter besides noradrenaline involved caused such response. Also, a study was done by Furness [31] on vas deferens from guinea pigs. Furness discussed the blockade failure of α-adrenoceptors antagonists for a couple of reasons: "Firstly, the probable inability of these agents to penetrate in sufficient concentrations to the neuromuscular junctions; secondly, their antagonism of the reuptake of noradrenaline into adrenergic axons; thirdly, their facilitation of transmitter release.” In the vas deferens of the rabbits and the guinea pigs, single sympathetic nerve stimulus results in a mechanical response which is very little or which may fail to happen completely, hence pulse trains that range from 2-32 Hz and which are used for 10-30 seconds are applied in the investigation of the neurogenic responses. In rats, there is a biphasic response to the pulse trains, with the initial peak starting within three to four seconds, later subsiding before the occurrence of the second phase, which reaches the peak within 10 seconds. Substantial evidence has also been provided by the antagonist studies, which suggest that the sympathetic response's initial phasic component gets mediated by the purine ATP while the second component gets mediated by noradrenaline.

Initial pharmacological evidence of cotransmission by purine ATP was found by studies that used photoaffinity label arylazidoaminopropionyl-ATP (ANAPP3), which was proven to be inhibiting the sympathetic contraction's initial phase in both the vas deferens and the contractions to the exogenous purine ATP. However, this did not affect the neurogenic response's second phase or the contractions from noradrenaline or even other agonists [32]. The results were confirmed by applying the table of properties of the P2-purinoceptors described by Burnstock et al. which delineates the ATP analogue, methylene-ATP, in the production of the selective desensitization of the P2 purinoceptors within the tissue. [33,34]. Mechanical response components are all stopped by the guanethidine and also the 6-hydroxydopamine, which cause selective destruction of the noradrenergic nerves, therefore showing that there is no releasing of ATP from the purinergic nerves’ distinct population. Reserpine, which depletes noradrenaline in noradrenergic nerves, inhibits the response's second component and not the beginning of phasic contraction [35].

Cotransmission in blood vessels

A variety of arteries sympathetic neurotransmissions tend to include ATP, noradrenaline, and a variety of peptides together with NPY. When arteries are considered, the muscle's electrical response to cotransmitters is very complicated compared to the vas deferens' response. Every sympathetic nerve stimulus forms a quick excitatory junctional potential (EJP) same in magnitude, pharmacological profile, and time course to the vas deferens. Nevertheless, when arteries are considered, pulse trains form slow depolarization with noradrenaline as the mediator. Noradrenaline and ATP contributions to the sympathetic vasoconstrictions greatly vary in every artery [26]. For instance, in the pulmonary artery of rabbits as well as the artery in the tail of rats, the ATP tends to have a very insignificant effect on sympathetic vasoconstriction. In the mesenteric arteries of both dogs and rats as well as the ileocolonic arteries of the rabbit, the biphasic response always has both noradrenergic as well as purinergic phases comparable to vas deferentia. In contrast, the small jejunal and saphenous arteries of the rabbit have vasoconstriction mainly through ATP mediation [36,37].

Apart from noradrenaline and ATP release, there is also the release of the NPY by the particular blood vessels or vas deferens sympathetic nerves. There needs to be more clarity on the functional role of NPY in the neurotransmission process. NPY also acts as a potent vasoconstrictor in some particular arteries, but in consideration of the low released concentrations, potentiating noradrenaline's constrictor effect is the action it has predominantly. It may also inhibit the release of noradrenaline by prejunctional action.

Some electrophysiological evidence for cotransmission

The first implementation of intracellular recording was done by Burnstock and Holman [38] on guinea pig vas deferens which opened the gate to study in-depth the sympathetic electro-activity in smooth muscle neurovascular junctions. After this EJPs, there was an assumption that noradrenaline, the discovered sympathetic nerves' transmitter, mediated them. It was two decades until a precious study made by Seddon and Westfall [39], who ran their experiments on the guinea pig vas deferens and showed that the contractions of it by nerve stimulation were biphasic. The idea that supported Westfall’s suggestion was a study done by Hogaboom and his colleagues early in the 80s [40] utilizing the purine ATP selective antagonists ANAPP3. Hogaboom’s study illustrated the relationship between ANAPP3 and adenine nucleotide in the guinea pig vas deferens by evaluation of the effect of ANPP3 on concentration-response relationships in the presence of light and in darkness. They came up with important findings such as ANAPP3 shifted concentration-response curve to the right and ANAPP3 was a non-competitive antagonist with visible light while it was a competitive antagonist in darkness. However, it was again identified in animals who were given various doses of reserpine where neuronal adrenaline was almost completely depleted. At the same time, there was a continuation of the ATP release, which led to EJPs not being reduced as seen under normal conditions [35].

Furthermore, vas deferens electrophysiological investigations have indicated that the reduction of EJP magnitude can be made by NPY through prejunctionally inhibiting the release of ATP [41]. The absence of NPY's selective antagonist creates difficulty in the confirmation of its function in the sympathetic vasoconstrictions. It has also currently been discovered that in some arteries of human beings that the vasoactive peptides, NPY with noradrenaline, are released from sympathetic nerves [42], while parasympathetic nerves secrete the vasoactive intestinal peptides (VIP), histamine isoleucine and acetylcholine [43]. Additionally, in the sensory nerves, calcitonin gene-related peptide (CGRP) is coreleased with the tachykinins [44]. These peptides seem to work like cotransmitters in both sympathetic as well as parasympathetic nerves, mainly at stimulation frequencies that are very high, with the stimulation in low frequencies releasing them from the sensory nerves [44].

Noradrenaline has very little contribution to the polarization of the membrane; the stimulation of the adrenoceptor tends to mediate neurogenic contraction's slow phase through the second messenger generation, which releases Ca2+ which is stored in the sarcoplasmic reticulum [45].

Cotransmission synergy

The response flexibility of the effector tissue could be enhanced by the presence of many transmitters, postjunctionally, since it is certain that various distinct transmitters may simultaneously relay various distinct messages to effector tissues. More common is the pattern where various substances work synergistically in enhancing the actions of others. Co-released substances can also work on both different target cells as well as the same target cells in tissues. For instance, the double action of the vasoactive intestinal peptide and the acetylcholine in the salivary glands forms vasodilation and stimulates the secretory cells. This indicated cotransmitters' functional synergism in the parasympathetic nerves [46].

Because every transmitter may activate a distinct effector pathway in the cell, the tachyphylaxis or desensitization, which may have occurred if one pathway was involved, gets avoided as a cotransmission consequence. For instance, the vascular smooth muscle contractions might be formed by Ca2+ released by the transmitter from the intracellular store, which provides stimulation to the production of the inositol triphosphate, or even by the influx of the Ca2+ through the openings of the ion channels, which have opened due to the activation of the receptor by various transmitters [45].

## Conclusions

It is clear that cotransmission is a key signaling mechanism by which neurons in all nervous systems operate. Work in multiple model systems, including those reviewed in this paper, has revealed some general concepts and supports a remarkable variety of mechanisms resulting from cotransmission. 
